# Infection-Induced Elevated Plasma Perampanel in a Patient with Hemimegalencephaly

**DOI:** 10.1155/2022/9844820

**Published:** 2022-04-29

**Authors:** Yuya Kinoshita, Hiroe Ueno, Hirofumi Kurata, Chizuru Ikeda, Erika Hori, Takumi Okada, Tomoyuki Shimazu, Isao Fujii, Makoto Matsukura, Hoseki Imamura

**Affiliations:** ^1^National Hospital Organization Kumamoto Saishun Medical Center, Department of Pediatrics, Koshi, Japan; ^2^Kumamoto University Hospital, Department of Pediatrics, Kumamoto City, Japan; ^3^Kumamoto Takumadai Rehabilitation Hospital, Department of Pediatrics, Kumamoto City, Japan; ^4^Aso Medical Center, Department of Pediatrics, Aso City, Japan; ^5^Kumamoto Kenhoku Hospital, Department of Pediatrics, Tamana City, Japan; ^6^Arao Municipal Hospital, Department of Pediatrics, Arao City, Japan; ^7^Kumamoto-Ashikita Center for The Severely Disabled, Department of Pediatrics, Ashikita, Japan

## Abstract

Perampanel is a noncompetitive, *α*-amino-3-hydroxy-5-methyl-4-isoxazolepropionic acid glutamate receptor antagonist. Herein, we report a case of increased perampanel plasma concentration and impaired consciousness triggered by an infection. The patient had refractory epilepsy associated with hemimegalencephaly. During adolescence, perampanel (maximum dose, 10 mg, oral), valproic acid, clobazam, and lacosamide were administered for seizure control. He was admitted to our hospital with high fever, impaired consciousness, and elevated perampanel plasma level (from 1,300 to 1,790 ng/mL), but with no increase in the concentration of other antiseizure medications. Further examinations (blood, cerebrospinal fluid, brain magnetic resonance images, and electroencephalogram) revealed no physical cause for impaired consciousness. After discontinuation of perampanel, his level of consciousness gradually improved. The pharmacokinetics of perampanel may be modified by both hemimegalencephaly and infection, resulting in an elevated plasma concentration of perampanel. This case underlines the importance of monitoring perampanel plasma concentration in patients with underlying brain disease who develop an infection.

## 1. Introduction

Perampanel is a novel, noncompetitive, *α*-amino-3-hydroxy-5-methyl-4-isoxazole propionic acid (AMPA) glutamate receptor antagonist that was approved in 2012 as an adjunctive therapy for refractory partial complex seizures. Perampanel was approved in Japan in March 2016 as a combination therapy for partial seizures and tonic-clonic seizures. Perampanel has a long half-life (105 h) and a large distribution volume (77 L/kg) [1]. Perampanel is metabolized in the liver, and thus, its plasma concentration increases in patients with liver damage [2]. The main side effects of perampanel include dizziness, drowsiness, and irritability; impaired consciousness is not a common side effect [3]. Of the few reports available regarding impaired consciousness due to perampanel, almost all were due to overdose [4–6]. Only one adult case was identified in which perampanel plasma concentration increased under appropriate oral administration due to an infection [7]. Herein, we report a pediatric case of increased perampanel plasma concentration, under appropriate oral administration, triggered by an infection, resulting in impaired consciousness.

## 2. Case Presentation

**Figure 1:**
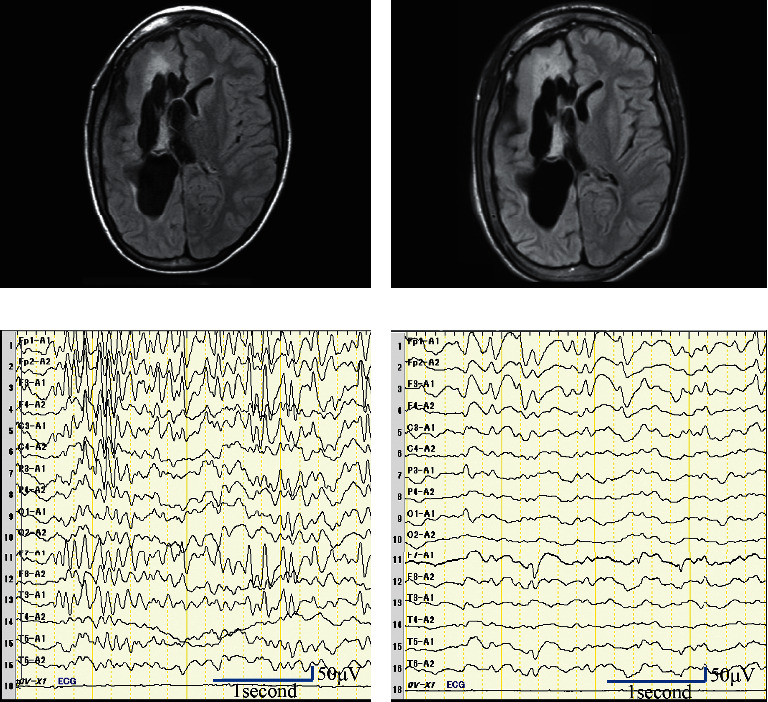
(a) MR image using fluid-attenuated inversion recovery (FLAIR) at 9 years 4 months. (b) Repeat FLAIR image obtained at 15 years 11 months (on day 7 after infection onset). (c) Electroencephalogram obtained during sleep at 15 years 1 month. (d) Electroencephalogram obtained during the period of impaired consciousness at 15 years 11 months. MR, magnetic resonance.

**Figure 2:**
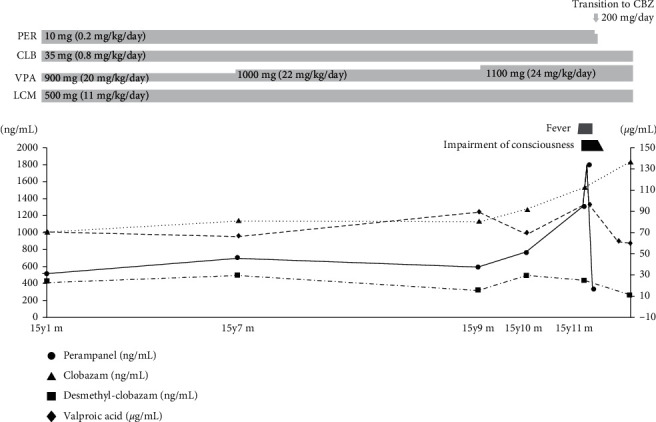
Clinical course. The patient's fever resolved immediately after hospitalization but with persistence of the state of impaired consciousness. Perampanel concentration was elevated at the time of admission (1,300 ng/ml) and ultimately increasing to a maximum of 1,790 ng/ml. Cessation of perampanel treatment improved the level of consciousness. Seizures increased with decreasing plasma concentration of perampanel. Fosphenytoin was initially introduced for seizure control, which was then switched to oral carbamazepine. The patient was discharged without apparent sequelae. VPA, valproic acid; CLB, clobazam; LCM, lacosamide; PER, perampanel; CBZ, carbamazepine.

The patient was a 15-year-old boy with refractory epilepsy associated with hemimegalencephaly. Interhemispheric transection was performed at 4 months of age for epilepsy treatment. At the age of 14 years and 8 months, perampanel treatment was initiated, in addition to valproic acid, clobazam, and lacosamide. Due to poor control of convulsions, the dose of oral perampanel was increased from 9 mg/day to 10 mg/day at the age of 15 years and 1 month, with monthly monitoring of the perampanel plasma concentration as per guidelines [8]. The perampanel plasma concentration before admission was as follows: 505 ng/mL (at 15 years and 2 months, 1 month after treatment initiation), 698 ng/mL (4 months before admission), 584 ng/mL (2 months before admission), and 758 ng/mL (1 month before admission). At 15 years and 11 months of age, the patient developed high fever due to a streptococcal infection. He was admitted to our hospital on day 6 after symptom onset, with fever of 39.4°C and impaired consciousness (Glasgow Coma Scale 6 (E1V1M4)). Meningitis, severe infection, and encephalopathy were considered as possible diagnoses. Blood samples indicated a slight decrease in white blood cell and platelet counts, deterioration of renal function (due to dehydration), and a slight increase in C-reactive protein level. There was no apparent liver damage. Glucose, sodium, potassium, calcium, vitamin B1, ammonia, and carnitine levels were normal (glucose, 99 mg/dL; sodium, 137 mg/dL; potassium, 4.03 mEq/L; corrected calcium, 9.0 mg/dL; vitamin B1, 43.2 ng/mL; ammonia, 58 *μ*g/dL; total carnitine, 67.2 *μ*mol/L; free carnitine, 52.2 *μ*mol/L; acyl carnitine, 15.0 *μ*mol/L; only the calcium concentration was measured on day 8). Cerebrospinal fluid (CSF) examination revealed no evidence of meningitis or acute disseminated encephalomyelitis (cells, 1 cell/mm^3^; protein, 33 mg/dL; and glucose, 60 mg/dL). Urinalysis, chest radiography, and electrocardiogram were normal. The electroencephalogram showed spikes and waves in the left frontal region, but the activity was suppressed compared to prior to disease onset (Figure 1). Brain computed tomography (CT) and magnetic resonance (MR) images did not show any remarkable changes. The patient was treated with cefotaxime and intravenous immunoglobulin (1.0 g/kg). The fever resolved within a day, although impaired consciousness persisted. On postadmission day 4 (day 9 after infection), a high perampanel plasma concentration was detected (1,300 ng/mL and 1,790 ng/mL on days 5 and 9, respectively). There was no increase in the plasma concentrations of other antiseizure medications (valproic acid, 97 *µ*g/mL; clobazam, 428 ng/mL; N-desmethylclobazam, 1,537 ng/mL; and lacosamide, 9.7 *µ*g/mL). The perampanel concentration of cerebrospinal fluid was 29 ng/mL. Therefore, an elevated perampanel plasma concentration was suspected as the cause of impaired consciousness. Perampanel was withdrawn and switched to oral carbamazepine. As the perampanel plasma concentration decreased, the patient could spontaneously open his eyes and speak and was able to independently assume a seated position. He was discharged on postadmission day 24 (29 days after infection onset) with no apparent sequelae (Figure 2).

## 3. Discussion

Perampanel is a novel antiseizure medication that was approved in the United States in 2012 and in Japan in 2016. A few previous reports have described impaired consciousness due to perampanel (Table 1), with overdose as a suicide attempt being the main cause of increased perampanel concentration. An amount of perampanel between 60 and 300 mg is considered as an overdose. This amount is associated with impaired consciousness, aggression, and cerebellar symptoms. The duration of impaired consciousness tends to be short, typically 1–4 days. Only one adult case was reported in which perampanel plasma concentration increased due to an infection under appropriate oral administration. In this adult case, the oral dose was 6 mg/day, with the perampanel plasma concentration increasing to 2,310 ng/mL after infection. In our case, the oral dose was 10 mg/day (under the threshold for overdose), and yet, the plasma concentration was >1,000 ng/mL, with impaired consciousness persisting for 8 days. The perampanel concentration in CSF was 29 ng/mL. Although above normal limits, this concentration was lower than the level previously reported as sufficient for coma induction [5]. Hence, we considered that the impaired level of consciousness in our patient was a result of brain anomalies associated with hemimegalencephaly.

We used the Naranjo scale to objectively evaluate whether the impaired consciousness of the patient in our case was induced by perampanel (Table 2). The Naranjo scale is a parameter first reported by Naranjo et al. in 1981 that assesses the causal relationship between drugs and clinically disturbing events [9]. In our case, the Naranjo scale score between perampanel and impaired consciousness was 7 points. On the Naranjo scale, a total score of 5–8 points was considered as being probable. Therefore, we presume that it is highly possible that perampanel contributed to impaired consciousness. The perampanel plasma concentration on day 5 was 1,300 ng/mL, and the perampanel plasma concentration on day 9 was 1,790 ng/mL. Since the perampanel plasma concentration increased sharply in 4 days, it is highly possible that it was not a chronic increase but rather an acute increase associated with fever. Before the patient was admitted this time, the dose of perampanel was fixed at 10 mg. The doses of clobazam and lacosamide did not change, and only the valproic acid dose was gradually increased (Figure 2). As valproic acid does not affect the perampanel plasma concentration [1], it is unlikely that the therapeutic regimen affected the perampanel plasma concentration. All antiseizure drugs were administered orally, and the route of administration for these drugs was not changed. In addition, the medical doctor and pharmacist monitored the daily medication. Hence, the observed elevation in the plasma perampanel concentration was not caused by the inappropriate administration of the medication.

The pharmacokinetics of antiseizure medications vary, with those for perampanel being unique. After oral administration, perampanel is absorbed from the intestinal tract at a rate of 100% and metabolized by the liver, mainly through cytochrome P450 3A4 (CYP3A4), leading to hepatic elimination (98%). The volume of distribution (Vd), half-life (*t*1/2), and protein binding rate are estimated at 1.1 L/kg, 52–129 h, and 95%, respectively [10]. Perampanel is not subjected to a first-pass effect nor does diet affect its bioavailability [11]. We note that an increase in perampanel plasma concentration can be caused by hepatic (but not renal) disorders [2]. In our case, liver dysfunction was not detected. However, inhibition of CYP3A4 can lead to an elevated perampanel plasma concentration [12]. CYP3A4 is known to be inhibited by azole antifungal drugs and macrolide antibiotics [12]. In addition to drugs, grapefruit juice inhibits CYP3A4 and St. John's wort induces the expression of the CYP3A4 enzyme [12]. In our case, with the exception of antiseizure drugs, the patient was taking only cefixime, tranexamic acid, and teprenone. We confirmed that the patient had not taken nonprescription supplements, such as herbs and grapefruit juice.

It is well documented that systemic infection or chronic inflammation may considerably alter the pharmacokinetics of many medications [13]. In our patient, a multidrug approach to seizure control was necessary, with plasma levels of valproic acid and clobazam controlled at a high level as medium concentrations were not effective. Long-term changes in plasma levels of valproic acid and clobazam were observable before illness, during hospitalization, and after discharge (Figure 2). After admission, we changed the patient's antiseizure medication from perampanel to carbamazepine. This led to a gradual decrease in perampanel plasma concentration and a consequent improvement in the patient's level of consciousness. Epileptic episodes were controlled after additional carbamazepine treatment. Clobazam is also metabolized by CYP3A4; however, the clobazam plasma concentration did not increase. A previous study reported that the concentration/dose ratio of perampanel increased when C-reactive protein level was high, with no effect on clobazam levels [14]. Clobazam is metabolized by CYP3A4, CYP2C19, and CYP2B6, which may explain why the plasma concentration of clobazam did not increase coincidentally with the increase in perampanel concentration. The Michaelis constant (Km) is a useful marker of plasma concentration of drugs that are metabolized by CYP3A4, with Km for clobazam being 29.0 [15]. Perampanel is a mild inducer of CYP3A4. Thus, although Km of perampanel is unknown, it appears to be higher than that of clobazam [1]. The relationship between the concentration/dose ratio of perampanel and CRP, however, is unclear, with reports of both increases and no causal relationship [14]. It has been reported that perampanel is metabolized by CYP1A2 and CYP2B6 [11]. If the actions of these metabolic enzymes could compensate for the action of CYP3A4, the plasma concentration of perampanel would have not increased. It has been reported that CYP3A metabolizes perampanel almost exclusively and that ketoconazole, a CYP3A4 inhibitor, actually increased the area under plasma concentration-time curve (AUC) of perampanel [16]. Ketoconazole is known to inhibit CYP3A4, but there are no reports demonstrating that ketoconazole induces the inhibition of CYP1A2 or CYP2B6. As CYP3A metabolizes perampanel almost exclusively, we hypothesized that CYP1A2 and CYP2B6 could not compensate for the action of CYP3A4 in the metabolism of perampanel. Similar to this report, it is possible that CYP1A2 and CYP2B6 could not be compensated for, as the perampanel plasma concentration in the patient in our case was elevated. It is reported that CYP2C19 and CYP2B6, in addition to CYP3A4, are important enzymes in clobazam and that CYP3A4 does not exclusively metabolize clobazam. Therefore, we considered that the actions of CYP2B6 and CYP3A4 could compensate for the action of CYP3A4 [15].

In our case, patient-specific factors, including structural brain abnormalities and hemimegalencephaly, might have contributed to impaired consciousness due to the increased plasma concentration of perampanel. Hemimegalencephaly has been associated with an abnormal mechanistic target of rapamycin (mTOR abnormality), such as a tuberous sclerosis complex [17]. On the other hand, inflammatory mediators are also said to have the potential to destroy the blood-brain barrier [18]. Therefore, in our patient, overactivation of proinflammatory signaling pathways could have increased the permeability of the blood-brain barrier. It may have been easily destroyed due to the patient's predisposition to hemimegalencephaly. However, as perampanel has a high protein binding affinity (>95%) to both albumin and alpha-1-acid glycoprotein [1] and considering the mild decrease in serum albumin concentration (minimum albumin, 3.0 mg/dL), it is unlikely that the plasma concentration of free perampanel increased.

The cause of impaired consciousness in our patient is difficult to clearly ascertain for several reasons. First, although the therapeutic reference range for perampanel is considered to be between 180 ng/mL and 980 ng/mL, with impaired consciousness occurring at levels >1,000 ng/mL, there are still no relevant data to support these reference values [19]. Moreover, therapeutic ranges usually differ across individuals and, thus, may not be necessarily consistent with a general reference range. Second, intravenous immunoglobulin (1.0 g/kg) was administered as acute encephalopathy could not be excluded. Last, other antiseizure medications may also contribute to impaired consciousness, for example, unbound valproic acid concentration might have interfered. Since the decrease in blood albumin concentration was mild in our patient (minimum albumin concentration, 3.0 g/dL), we speculate that the concentration of unbound valproic acid might not have increased significantly. However, we note that we were unable to measure the concentration of unbound valproic acid at that time. On the other hand, plasma levels of other antiseizure medications were also controlled at a high level, which may have contributed to impaired consciousness. But perampanel has clearly changed plasma levels, and we consider that perampanel is the medication that contributed most to impaired consciousness.

In conclusion, we report a case of increased perampanel plasma concentration triggered by infection which led to impaired consciousness in an adolescent with hemimegalencephaly. The increase in perampanel plasma concentration was triggered by inflammation and further influenced by hemimegalencephaly. Therefore, perampanel plasma concentration should be monitored, especially in patients with an underlying brain disease who develop an infection. Other antiseizure medications may also have contributed to impaired consciousness, so be careful when using perampanel in combination with multiple medications. Accumulation of cases will be important to improve our understanding of the factors influencing perampanel plasma concentration with infections.

## Figures and Tables

**Table 1:** Case reports of impaired consciousness due to perampanel.

Case	Age (years)	Sex	Dose	Underlying disease	Trigger	Period of impaired consciousness (days)	Outcome
Li et al., 2018 [5]	54	Male	300 mg (once)	Refractory epilepsy	Overdose	4	He had cerebellar symptoms after awakening, but recovered spontaneously.
Wu et al., 2019 [4]	40	Male	60 mg (once)	Depression History of overdose	Overdose	1	He self-extubated after mechanical ventilation was administered.
							He was aggressive after waking and was sedated for 5 days.
							He had irritability and anxiety until the 9th day.
Hoppner et al., 2013 [6]	34	Female	204 mg (once)	Partial epilepsy Tuberous sclerosis	Overdose	2	She was disorientated after awakening, but recovered spontaneously.
Mutata and Nobukuni, 2020 [7]	74	Female	6 mg (every day)	Temporal epilepsy	Infection	Not provided	She was treated with an antimicrobial infusion and her consciousness gradually improved.
Our patient	15	Male	10 mg (every day)	Hemimegalencephaly Refractory epilepsy	Infection	8	He recovered after IVIG and tapering off perampanel treatment.

IVIG, intravenous immunoglobulin.

**Table 2:** Evaluation of the causal relationship between perampanel and impaired consciousness using the Naranjo scale.

	Yes	No	Do not know	Score
1. Are there previous conclusive reports on this reaction?	+1	0	0	+1
2. Did the adverse event appear after the suspected drug was administered?	+2	−1	0	+2
3. Did the adverse reaction improve when the drug was discontinued or a specific antagonist was administered?	+1	0	0	+1
4. Did the adverse reaction reappear when the drug was readministered?	+2	−1	0	0
5. Are there alternative causes (other than the drug) that could on their own have caused the reaction?	−1	+2	0	+2
6. Did the reaction reappear when a placebo was given?	−1	+1	0	0
7. Was the drug detected in blood (or other fluids) in concentration known to be toxic?	+1	0	0	+1
8. Was the reaction more severe when the dose was increased or less severe when the dose was decreased?	+1	0	0	0
9. Did the patient have a similar reaction to the same or similar drugs in any previous exposure?	+1	0	0	0
10. Was the adverse event confirmed by any objective evidence?	+1	0	0	0
			Total score	+7

Total score ≥9, definite ADR; 5–8, probable ADR; 1–4, possible ADR; 0, doubtful ADR. ADR, adverse drug reaction.
